# An Unusual Mishap during Root Canal Access in Retreatment Case

**DOI:** 10.1155/2012/892652

**Published:** 2012-10-02

**Authors:** Manjunath Hampanna Malur, Akash Krishna, D. V. Sapna

**Affiliations:** ^1^Department of Conservative and Endodontics, Peoples College of Dental Sciences & Research Centre, Bhanpur, Bhopal 462037, India; ^2^Department of Conservative and Endodontics, Dayanand Sagar Dental College, Bangalore 560001, India

## Abstract

Varieties of objects have been introduced into the root canal system accidentally or intentionally; removal of these objects necessitates the success of the treatment. This paper explains an unusual bur fracture during nonsurgical endodontic treatment and its removal by ultrasonic energy with ease and without the removal of extra large amount of root dentin.

## 1. Introduction

During preparation of the root canal system in root canal treatment, there is inclusion of many objects accidentally or intentionally, either by the dentist or by patients. The removal of these objects always necessitates the success of the treatment.

Some of the items, like obturating materials or posts, are introduced intentionally, whereas many others are introduced inadvertently. Successful orthograde retrieval of intracanal obstruction may be difficult but essential if nonsurgical retreatment is necessary. 

Iatrogenic inclusion of various articles into root canals has been reported. These include absorbent points [1], burs [2, 3], files [4–6], glass beads, and amalgam or gold fillings. In addition, patients may block root canals in teeth that have been left open to drain. Attempted removal of these foreign objects has led to the development of several devices, instruments, and techniques which aid in the retrieval of canal obstructions. Most of them include the various devices like broach wrapped in cotton for removal of loose fragments. Another method is to prepare a trench around the item with a half-round bur and then to use splinter forceps. Rasps for removal of large fragments, use of Gates Glidden drills, trepan burs, and extractors aided fiber optic were used to remove items. They stated that canal must be straight and wide enough to accommodate the rigid extractor. However, most of these procedures are tedious procedures and lead to removal extralarge amount of root dentin and may lead to gouging or perforation of the root.

 Most recently, ultrasonics has been utilized to remove solid objects from the pulp space [7, 8] for removal of cemented silver cones or endodontic instruments with a cavitron tip. In this paper, we have used endosonics for the removal of broken bur inside root canal system.

## 2. Case Report

A 20-year-old female patient reported the chief complaint of discolored 21. The patient has the history of trauma with upper front tooth region around 10 years before. The patient has the history of orthodontic treatment around 5 years before. History of present illness reported that the patient has undergone root canal treatment 2 months before. Radiograph with 21 reveals well-circumscribed lesion around 21 and 22. Nonsurgical endodontic treatment was planned for the patient.

**Figure 1:**
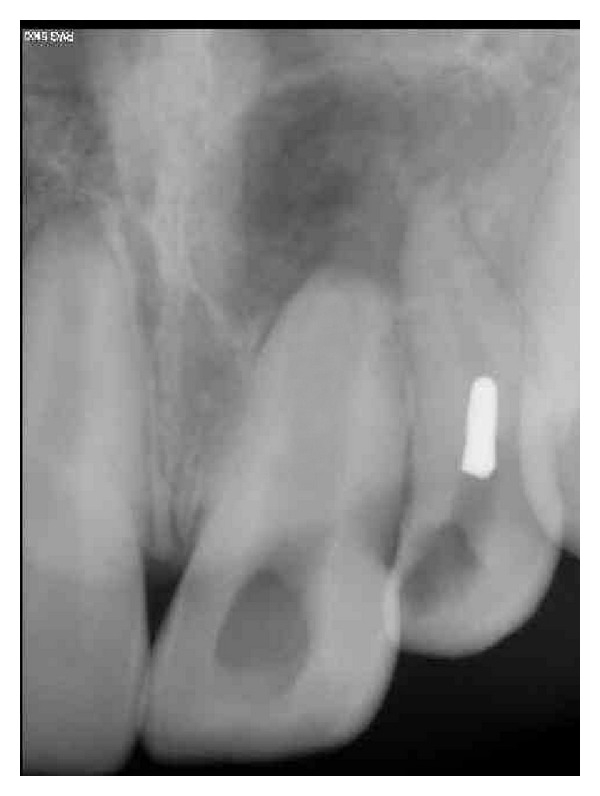
Bur fractured at middle third of the canal.

 Local anesthesia was given with 22, rubber dam applied, GP retrieval done with 21, and access cavity initiated with 22. During access cavity refining with 22 by carbide bur, the bur fractured. Attempted removal of bur by using H-file further pushed the bur into the middle third of the root. Since the nonsurgical endodontic treatment was planned for the patient, the orthograde removal of bur necessitated the completion of the treatment. Radiograph was taken with 22 revealing that bur was fractured in the middle third (Figure 1), and curvature at the middle third of the root prevented the further pushing of the bur apically. Moreover, radiograph revealed the improper tapering of the canal that made bur to occlude and led to the fracture. 

**Figure 2:**
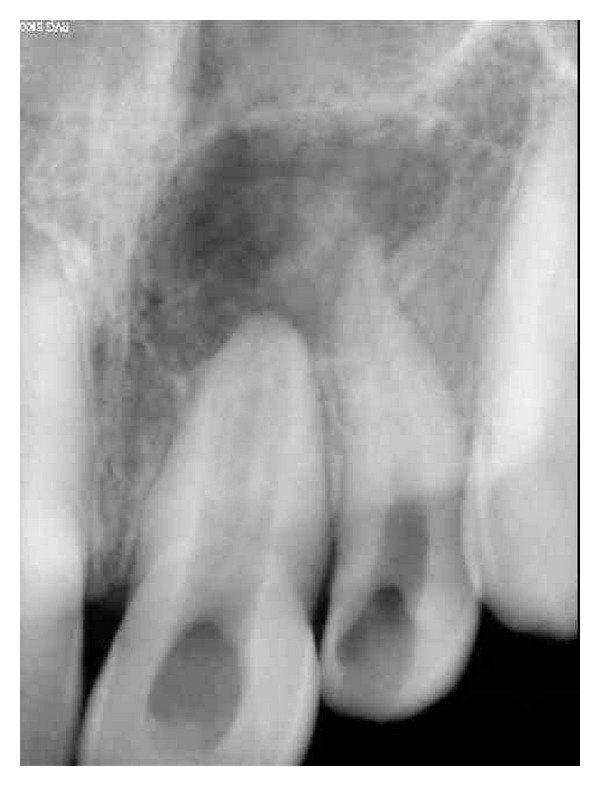
Radiograph revealing the removal of broken bur, also note the coronal flaring that allowed the easy removal of the bur fragment.

Magnifying loupes were used to observe the canal, and initial alteration in access cavity was made so that the bur can easily come out of the canal without holding anywhere after the ultrasonic forces were applied. Radiograph revealed the lateral space distolabially, and the Cavi-Endo ultrasonic tip was then placed directly in contact with the object to be removed at distolabial area until the ultrasonic vibration loosened the object. Another little space was made opposite to the distolabial area, that is, mesiolingually between the bur and the root dentin by using Gates Glidden drill number 2, so that the ultrasonic tip was placed easily on that area. The ultrasonic tip was kept and operated causing slow movement and slipping of the bur from the occluded portions. By the altered coronal tapering (Figure 2) of the canal, the bur was easily removed outside the canal. 

**Figure 3:**
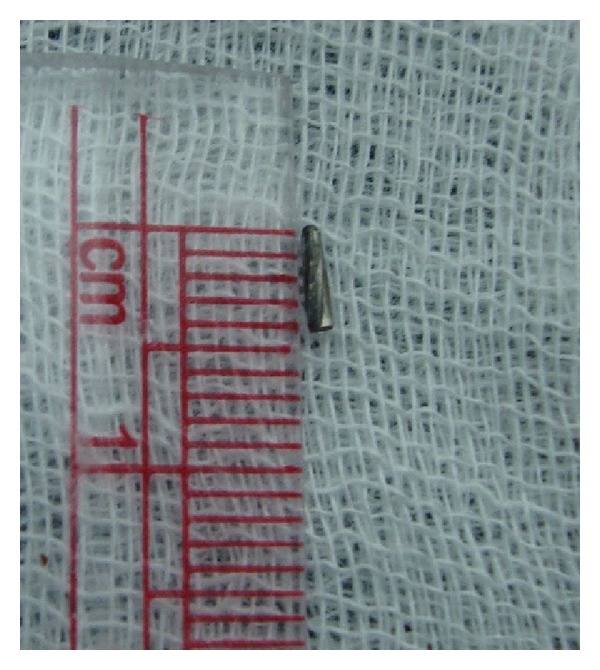
Broken bur fragment measuring about 4 mm.

**Figure 4:**
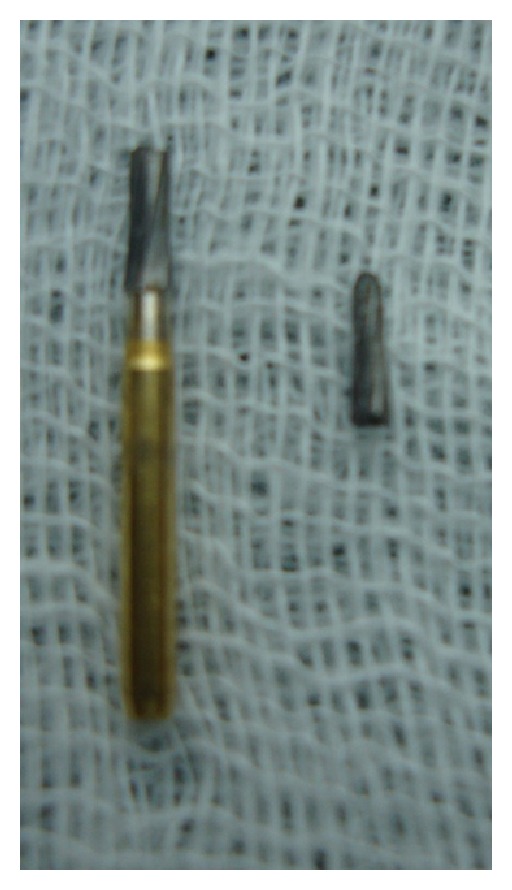
Broken bur with its fragment.

The broken bur was measuring about 4 mm (Figures 3 and 4). Biomechanical preparation was done with both 21 and 22, polyantibiotic paste, and chlorhexidine medicament given with both, respectively, for 10 days, rendering the tooth symptomless. After canal rendered painless, calcium hydroxide medicament was given for both 21 and 22 and monitored for its progress of the treatment.

## 3. Summary

A large number of miscellaneous items either intentionally or otherwise find their way into root canals. Various retrieval techniques, many of which involve excessive removal of dentin, have evolved. Endosonics has been found to be a very useful adjunct in the retrieval method. Its major advantage is that it, in many cases, enables retrograde (nonsurgical) removal of canal obstructions without weakening teeth by excessive dentin removal. We have been successful in attempts to remove difficult obstructions in many but not all cases. Some items are easily and quickly removed; others require more time and patience. Endosonics alone or in combination is very useful in the retrieval of canal obstructions.

## References

[1] Grossman LI (1974). Endodontic case reports. *Dental Clinics of North America*.

[2] Sternberg RN (1977). Retrieval of broken instrument from root canal. *Oral Surgery, Oral Medicine, Oral Pathology*.

[3] Meidinger DL, Kabes BJ (1985). Foreign object removal utilizing the Cavi-Endo ultrasonic instrument. *Journal of Endodontics*.

[4] Fors UGH, Berg JO (1983). A method for the removal of broken endodontic instruments from root canals. *Journal of Endodontics*.

[5] Souyave LC, Inglis AT, Alcalay M (1985). Removal of fractured endodontic instruments using ultrasonics. *British Dental Journal*.

[6] Feldman G, Solomon C, Notaro P, Moskowitz E (1974). Retrieving broken endodontic instruments. *The Journal of the American Dental Association*.

[7] Faramarzi F, Fakri H, Javaheri HH (2010). Endodontic treatment of a mandibular first molar with three mesial canals and broken instrument removal. *Australian Endodontic Journal*.

[8] Gencogla N, Hetracioglu D (2009). Comparison of the different techniques to remove fracture endodontic instruments from root canal systems. *European Journal of Dentistry*.

